# Amniotic Band Syndrome—Prenatal Diagnosis

**DOI:** 10.3390/diagnostics14010034

**Published:** 2023-12-23

**Authors:** Nicolae Gică, Livia Mihaela Apostol, Corina Gică, Iulia Huluță, Ana Maria Vayna, Anca Maria Panaitescu, Nicoleta Gana

**Affiliations:** 1 Gynecology Department, Faculty of Medicine, Carol Davila University of Medicine and Pharmacy, 020021 Bucharest, Romania; gica.nicolae@umfcd.ro (N.G.); mat.corina@gmail.com (C.G.); iuliahuluta16@gmail.com (I.H.); anamariavayna@gmail.com (A.M.V.); anca.panaitescu@umfcd.ro (A.M.P.); gana_nicoleta@yahoo.com (N.G.); 2 Clinical Hospital of Obstetrics and Gynaecology Filantropia, 011171 Bucharest, Romania

**Keywords:** amniotic band syndrome, body stalk anomaly, abnormal fetal extremities

## Abstract

This is a case of a fetus affected by an amniotic band detected at 20 weeks of gestation. A presumptive diagnosis was made based on the ultrasound features. The ultrasound showed an abnormally developed right lower limb and no other associated fetal abnormalities. The unilaterality of the defect decreases the chances of genetic abnormality or an early vascular insult. The postnatal examination of the newborn concluded that the prenatal diagnosis was right.

Amniotic band syndrome is a condition that refers to a spectrum of fetal anomalies caused by a fibrous amniotic band. It can involve the extremities, the cranium and face, the trunk and the abdomen. The syndrome can present in isolation or in any combination, with the end of the spectrum being a body stalk anomaly. The ultrasound diagnosis is based on visualizing the consequences of the amniotic band; most of the time, the amniotic band cannot be demonstrated by ultrasound. Most frequently, the parts involved are the extremities, causing the absence of digits, amputations or abnormally developed extremities. We could also see facial clefts, encephalocele, spinal defects, abdominal wall anomalies or underdeveloped organs. There is no increased risk of genetic or chromosomal abnormalities. The incidence of amniotic band syndrome is thought to be one in twelve hundred births [1]. The etiology in not understood, but an early amnion rupture in pregnancy can lead to multiple bands adherent to the fetus [2]. The prognosis depends on the spectrum of manifestations from minor to lethal abnormalities. The follow-up should include detailed ultrasound examination every 2–3 weeks to evaluate the progression on the affected part of the fetus and a detailed fetal echocardiography [3,4]. In some isolated cases where there is a risk of amputation or constriction of the umbilical cord, the fetoscopic release of the constriction ring can be attempted. If the pregnancy continues, the delivery place should have a facility for neonatal intensive care [3].

We present a case of a 30-year-old woman who presented at 25 weeks of pregnancy for a second opinion due to a fetal right lower limb deformity detected at 20 weeks gestation. This was her second ongoing pregnancy following spontaneous conception. She previously delivered by CS a full-term baby with no significant history. She did not perform the first trimester ultrasound examination and the screening for chromosomal abnormalities. The triple test showed a low risk for Down’s syndrome. No remarkable family or personal history was noted.

Our ultrasound examination revealed a normally grown female fetus, with an abnormal development of the right lower extremity. The right femur measured within normal ranges for the gestational age. There were flexion/extension movements at the level of the knee joint. Just below the right knee, the right leg appeared shorter and thinner than the contralateral one. The tibia and fibula from the right shin were below the third centile, with a mild curvature (see Figure 1a). Above the right knee, at the level of the right thigh, there was skin oedema. The right foot was shorter, and it had an abnormal shape (see Figure 1b). The rest of fetal anatomy appeared unremarkable, and in particular, the spine, ribs, upper extremities and left lower extremity had no additional changes.

We explained to the parents that this anomaly was isolated. Because of the unilaterality of the defect, it was less likely that this was associated with underlying chromosomal abnormalities or genetic syndromes. The amniocentesis results showed a normal karyotype and normal array CGH. Our diagnosis was amniotic band syndrome. This is a rare finding when a fibrous amniotic band entangles parts of the fetus and constrict the blood supply to a specific part and causes the deformity of different parts of the fetus (see Figure 2).

We followed the growth of the fetus and any additional changes in the right leg abnormality every 2 to 3 weeks. Fetoscopic intervention was not suitable in this case because the right leg continued to grow below the third centile, and there was not any suspicion of progression towards the amputation of the right leg.

A differential diagnosis can, however, be made with a vascular insult in the very early stages of development of the embryo, but this is usually bilateral.

The baby was delivered at 39 weeks by CS, and they were a 3300 g female newborn. In addition to the ultrasound findings, they were missing nails on the contralateral foot (see Figure 3a). The surgical and genetic examinations after birth concluded that this abnormality was caused by an amniotic band. The baby had two surgeries after birth in order to improve the outcome of the right leg, and the orthopedic team discussed the possibility of a prosthetic aid for a reasonably functional result (see Figure 3b).

In conclusion, there is a high level of variability in the manifestations of this condition, and the outcome is dependent on the extension of the lesion. From a neurological point of view, in cases with isolated abnormalities of the extremities, the prognosis is good. Advanced plastic and orthopedic surgery can provide a good cosmetic solution and restore the function of the affected leg.

## Figures and Tables

**Figure 1:**
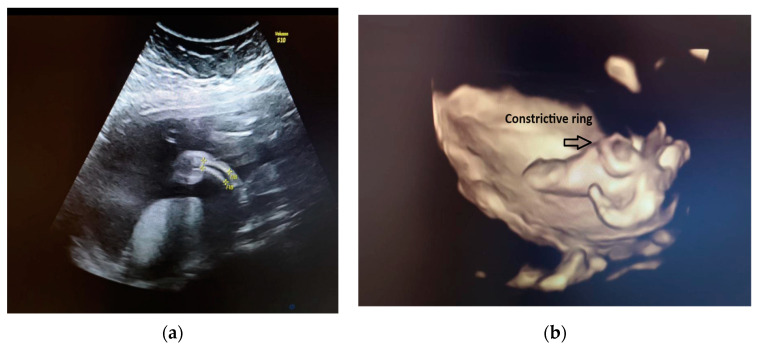
(**a**) Fetal right leg; (**b**) 3D reconstruction of the fetal right leg.

**Figure 2:**
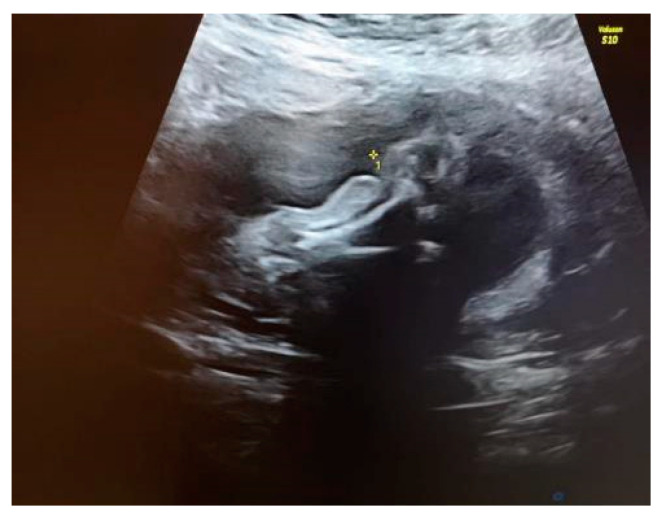
Visualization of the constricted area of the right shin.

**Figure 3:**
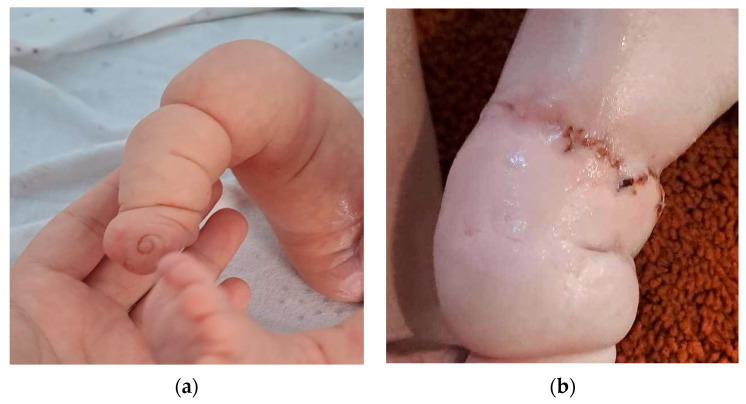
(**a**) Postnatal aspect of the right lower limb. (**b**) Right lower limb after surgery.
